# Editorial: Lymphocytes in MS and EAE: More Than Just a CD4^+^ World

**DOI:** 10.3389/fimmu.2017.00133

**Published:** 2017-02-13

**Authors:** Manu Rangachari, Steven M. Kerfoot, Nathalie Arbour, Jorge Ivan Alvarez

**Affiliations:** ^1^Department of Neurosciences, Centre de recherche du CHU de Québec – Université Laval, Quebec City, QC, Canada; ^2^Department of Molecular Medicine, Université Laval, Quebec City, QC, Canada; ^3^Department of Microbiology and Immunology, Schulich School of Medicine and Dentistry, Western University, London, ON, Canada; ^4^Department of Neurosciences, Université de Montréal and CRCHUM, Montréal, QC, Canada; ^5^Department of Pathobiology, University of Pennsylvania, Philadelphia, PA, USA

**Keywords:** multiple sclerosis, experimental autoimmune encephalomyelitis, CD4^+^ T cell, CD8^+^ T cell, B cell, gamma-delta T cell, MAIT cell, NK cell

**Editorial on the Research Topic**

**Lymphocytes in MS and EAE: More Than Just a CD4+ World**

Multiple sclerosis (MS) is an autoimmune disease of the central nervous system (CNS) that affects nearly two million people worldwide. Disease onset can occur at a young age, leaving sufferers with a significantly reduced quality of life. The fact that there is no cure for MS, plus the fact that its animal model experimental autoimmune encephalomyelitis (EAE) is considered a “classic” model of CD4^+^ T cell-triggered autoimmunity, has led MS pathogenesis to be an area of intense investigation in the past few decades. Numerous lines of evidence indicate that MS is driven by CD4^+^ T lymphocyte-mediated mechanisms (1–3). Polymorphisms in the HLA class II region are by far the strongest genetic link to MS (4). Moreover, the majority of currently available MS disease-modifying therapies are believed to act by modulating inflammatory CD4^+^ T cell responses, although in many cases the effects on other immune cell types are under-studied and may be at least as important. Furthermore, while current immunomodulatory drugs are able to reduce the frequency and severity of MS relapses, they are relatively ineffective in progressive forms of the disease (5). Attempted blockade of CD4^+^ T cells using anti-CD4-depleting antibody therapy did not produce clinical benefits to patients with MS (6). In contrast, more global immunosuppressive or immunomodulatory approaches reduce the number of relapses and disease progression of MS patients (7). Furthermore, numerous publications have presented data generated from autopsy material obtained from human patients supporting the notion that other cell types are involved (8–11). There is thus an urgent need to expand our view of immune-related pathogenesis in human disease. Despite evidence that lymphocytes such as B cells and CD8^+^ T cells play a role, their contributions are much less well studied experimentally compared to those of CD4^+^ T cells. In this Special Topic, we promote this expanded view of disease pathogenesis by presenting articles that examine the role played by lymphocytes other than CD4^+^ T cells in MS and its experimental models.

The B cell-depleting reagents rituximab and ocrelizumab have shown success against relapsing/remitting (12) and even progressive MS (13). Thus, it is no surprise that this collection features several submissions considering different aspects of potential B cell contributions to CNS autoimmunity. Both Claes et al. and Michel et al. survey what is known about B cells in MS and discuss potential pathogenic mechanisms including antibody production, cytokine secretion, antigen presentation to T cells, and the promotion of disease from within the CNS. Claes et al. additionally present a detailed review of how B cell subpopulations and effector functions are altered both by broadly specific disease-modifying therapies such as interferon-beta and glatiramer acetate as well as by rituximab and ocrelizumab. B cell infiltration into the CNS in MS was a particular focus of the Michel et al. review, and this was further expanded upon by two additional reviews in this special topic. Blauth et al. describe the signals that may permit B cell entry to the CNS, such as CXCL13, VLA-4, and ICAM. Additionally, they discuss recent evidence suggesting that B cells can also exit the CNS so as to undergo additional affinity maturation in peripheral lymphoid tissues, as well as data indicating that the meninges can support differentiation of CNS-specific B cells independently of the periphery. Once in the CNS, B cells have been described to form aggregates in the meninges akin to tertiary lymphoid organ-like structures, and these are the subject of a review from Pikor et al. In particular, they discuss recent evidence suggesting that antigen-experienced T and B cells accumulate in these structures to promote CNS inflammation.

A common theme of these reviews is the uncertainty regarding the neuropathogenic role of B cells in MS. Interestingly, evidence from the two primary research articles in this issue present a challenge to the hypothesis that autoreactive B cell responses are propagated within meningeal aggregates of lymphocytes. First, in an attempt to identify antigenic targets of B cell-mediated destruction in MS, Willis et al. cloned the IgV heavy and light chains of CNS-infiltrating B cell clones from six MS patients to generate putative CNS-reactive recombinant antibodies. Surprisingly, using various approaches (binding to candidate antigen, CNS cell lines, or antigen array) no CNS- or MS-specific antigen targets could be identified. Second, Dang et al. present data showing that the presence of B cells in spinal cord-associated meningeal clusters correlates with chronic symptoms in a B cell-dependent model of spontaneous EAE. Intriguingly, however, these “cluster B cells” have a naïve phenotype with little evidence of Ig class switching and the clusters themselves do not bear the features of structured lymphoid follicles, suggesting that the simple presence of B cells in the meninges is sufficient to promote disease progression.

The remaining B cell-centric articles in this issue focus on other potential pathogenic mechanisms. B cells almost certainly shape the autoimmune response through the secretion of inflammatory cytokines such as TNFα, lymphotoxin, and GM-CSF, as described by Li et al. In addition, B cells can present antigen to T cells and modulate their properties. Márquez and Horwitz discuss the possibility that Epstein-Barr virus (EBV)-infected B cells preferentially elicit Th1 responses in the CNS. It is tempting to speculate that exposure to EBV, which is an environmental factor strongly associated with MS (14), influences disease outcomes by altering B cell activity. Furthermore, the effectiveness of B cell depletion therapy in MS may be due in part to reduced effector T cell function. While the “immune helper” functions of B cells in MS have been intensely investigated, the classic role of B cells as antibody-secreting cells cannot be neglected. Indeed, the presence of oligoclonal IgG bands in CSF has long been a clinical biomarker of MS and is still used as a differential diagnostic tool (15). Khorooshi et al. detail what is known about antibody-mediated pathogenic mechanisms in CNS autoimmunity. They pay particular attention to the role of anti-aquaporin 4 antibodies in neuromyelitis optica—a disease that has only recently been recognized as being independent of MS. They present a scheme in which these antibodies cross a disrupted blood–brain barrier and target astrocytes for complement-mediated destruction in a T cell-independent manner.

CD8^+^ T cells are present in MS tissue at all stages of disease. They can greatly outnumber CD4^+^ T cells in lesions, perivascular cuffs, and normal-appearing white matter. Furthermore, unlike CD4^+^ T cells that mostly remain restricted to the perivascular space, CD8^+^ T cells infiltrate deep into the CNS parenchymal lesions (16). Salou et al. delineate some of the current lines of investigation into the role of CD8^+^ T cells in MS, such as ongoing efforts to characterize the antigenic repertoire of these cells, as well as recent advances in the study of CD8^+^ T cells using EAE and the importance of IL-17-producing CD8^+^ T cells in pathogenesis. Yang et al. describe the pathogenic function of CD8^+^ T cells in peripheral neuropathies such as Guillain–Barré syndrome, with a particular emphasis on a mouse model that features CD8^+^ T cell-driven inflammation in the peripheral sciatic, trigeminal, and facial nerves. By contrast, Sinha et al. argue that the role of regulatory CD8^+^ T cells (T_reg_) in CNS autoimmunity deserves further attention. Importantly, the frequency of CD8^+^ T_reg_ is diminished during MS relapse, and the authors discuss findings suggesting that the drug glatiramer acetate may act, in part, by augmenting the CD8^+^ T_reg_ response. Finally, Ignatius Arokia Doss et al. describe a transgenic mouse strain (1C6) in which both CD4^+^ and CD8^+^ T cells bear T cell receptor specificity to myelin antigen. The 1C6 transgene is on the NOD background, on which mice develop a relapsing-to-progressive pattern of EAE that models the form most commonly seen in human MS. The authors propose an approach in which 1C6 CD8^+^ T cells are stimulated *ex vivo* with distinct differentiation stimuli, prior to adoptive transfer. This would allow one to dissect the relative contributions of IFNγ-producing, IL-17-positive, and potentially even regulatory CD8^+^ T cells to CNS autoimmunity.

While B cells and CD8^+^ T cells represent two-thirds of the lymphocytic “Holy Trinity,” there is an array of other lymphocyte subsets with innate immune-like properties that have been posited to play a role in MS. Edwards et al. and Malik et al. provide an overview of what is known about γδ T cells in MS and EAE, respectively. These cells, which are found principally in skin and mucosal tissues, readily produce IL-17-associated cytokines and thus may be important mediators of inflammation in the CNS. The role of natural killer cells in disease may be Janus-like, with inflammatory CD16^+^CD56^dim^ cells being increased during MS relapse while the CD16^dim/-^CD56^bright^ subset predominates during remission (Edwards et al.). Treiner and Liblau report what is known about recently identified mucosal-associated invariant T (MAIT) cells. These are lymphocytes with innate properties that, as their name suggests, are located in mucosal tissues such as in the gut. While studies in mouse EAE have suggested that MAIT cells are anti-inflammatory, the picture is less clear in humans as MS immunotherapy appears to affect the frequency of peripheral MAIT cells. However, as MAIT cells may proliferate in response to commensal microbial antigens (17), it is tempting to speculate that they may provide part of the answer to the question of how the microbiome can influence autoimmune inflammatory diseases such as MS.

The past decades have seen remarkable progress in unraveling the complex and diversified immune mechanisms that contribute to MS pathobiology. Nevertheless, the precise etiology of MS remains elusive. Furthermore, despite an increasing number of immunomodulatory or immunosuppressive therapies altering relapsing-remitting MS, there is a pressing need for effective treatments for progressive disease. The more expanded view of disease pathology presented in this Special Topic may prove to be the key for the next generation of MS therapies.

## Author Contributions

MR wrote the manuscript with the input of SK, NA, and JA.

## Conflict of Interest Statement

The authors declare that the research was conducted in the absence of any commercial or financial relationships that could be construed as a potential conflict of interest.
